# Synthesis and Protective Effect of New Ligustrazine-Benzoic Acid Derivatives against CoCl_2_-Induced Neurotoxicity in Differentiated PC12 Cells

**DOI:** 10.3390/molecules181013027

**Published:** 2013-10-18

**Authors:** Penglong Wang, Honggui Zhang, Fuhao Chu, Xin Xu, Jinxuan Lin, Chunxiao Chen, Guoliang Li, Yatao Cheng, Lin Wang, Qiang Li, Yuzhong Zhang, Haimin Lei

**Affiliations:** 1 School of Chinese Pharmacy, Beijing University of Chinese Medicine, Beijing 100102, China; E-Mails: wpl581@126.com (P.W.); bzy714@163.com (H.Z.); chufhao@163.com (F.C.); xuxin_asia@163.com (X.X.); Liguoliangsx@163.com (G.L.); artery86@hotmail.com (Y.C.); wanglin431@126.com (L.W.); lq_cn@126.com (Q.L.); 2 Department of Pathology, Beijing University of Chinese Medicine, Beijing 100102, China; E-Mails: mmzmmk@163.com (J.L.); springxiao-85@163.com (C.C.)

**Keywords:** ligustrazine, neuroprotective effect, cobalt chloride, PC12 cell, HE staining

## Abstract

A series of novel ligustrazine-benzoic acid derivatives were synthesized and evaluated for their protective effect against cobalt chloride-induced neurotoxicity in differentiated PC12 cells. Combining hematoxylin and eosin staining, we found compound that (3,5,6-trimethylpyrazin-2-yl)methyl 3-methoxy-4-[(3,5,6-trimethylpyrazin-2-yl)methoxy]benzoate (**4a**) displayed promising protective effect on the proliferation of the injured PC12 cells (EC_50_ = 4.249 µM). Structure-activity relationships are briefly discussed.

## 1. Introduction

Stroke is one of the most devastating diseases in China as well as worldwide. Most strokes (80%) are ischemic and the disease relates with both cerebrovascular system and cranial nerves [1]. Currently, treatments for ischemic stroke include the use of thrombolytic and neuroprotective agents; thrombolytic drugs lyse blood clots to restore blood flow, and neuroprotective treatments prevent cell death during and after ischemia and reperfusion [2,3]. Despite the remarkable progress achieved in the therapy of stroke during the last two decades, there is no effective chemotherapy for cerebral ischemic stroke and neuroprotective agents that could attenuate lots of the clinical problems of ischemic stroke [1,4], so the development of new neuroprotective agents that inhibit neuronal damage induced by cerebral ischemia is of great significance [4].

Ligustrazine (2,3,5,6-tetramethylpyrazine, TMP), one of the major effective components of the Chinese traditional medicinal herb *Ligusticum Chuanxiong Hort*, has been widely used for ischemic stroke therapy in China for many years. TMP has multiple mechanisms of action, including thrombolysis and neuroprotection, both of which were important for effectively protecting the brain tissue from ischemic and reperfusion damage in ischemic stroke [5,6,7,8]. A series of previous studies proved that ligustrazinyloxy-aromatic acid derivatives could inhibit platelet aggregation and protect damaged endothelial cell proliferation, and that they exhibited much higher activities than TMP [9,10,11]. Many of these aromatic acids, such as protocatechuic acid, vanillic acid, salicylic acid, the parent compounds of ligustrazine derivatives, also showed interesting neuroprotective activity [12,13,14,15,16]. To further improve TMP’s neuroprotective effects, inspired by the biological characteristics of TMP and aromatic acids, we integrated the ligustrazine ring and hydroxybenzoic acid fragments into one molecule according to the combination principle in medicinal chemistry, constructing a series of new ligustrazine-benzoic acid derivatives.

PC 12 is a cell line derived from a rat adrenal medulla pheochromocytoma. The differentiated PC12 cells induced by nerve growth factor (NGF) have the typical characteristic of the neurons in form and function; therefore it is widely used as a model for *in* *vitro* neuron research [17,18]. Cobalt chloride (CoCl_2_), a water-soluble compound, was used in this investigation because it is one of the best-known chemical inducer of PC12 injury; CoCl_2_-induced neurotoxicity in differentiated PC12 cells is commonly used to screen new candidates for the intervention of stroke [19,20]. Herein, the novel ligustrazine-benzoic acid derivatives’ neuroprotective activities were evaluated on CoCl_2_-induced damage in differentiated PC12 cells by 3-(4,5-dimethylthiazol-2-yl)-2,5-diphenyltetrazolium bromide (MTT) assay and hematoxylin and eosin (HE) staining. Structure-activity relationships (SARs) of these new compounds are also discussed.

## 2. Results and Discussion

### 2.1. Chemistry

**Scheme 1:**
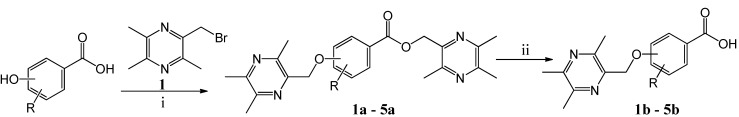
Synthetic routes to ligustrazine derivatives **1a**–**5a**, **1b**–**5b**.

**Scheme 2:**
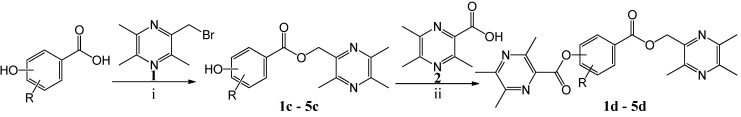
Synthetic routes to ligustrazine derivatives **1c**−**5c**, **1d**−**5d**.

All the designed derivatives were synthesized via the routes outlined in Scheme 1 and Scheme 2. As the important intermediates, 2-(bromomethyl)-3,5,6-trimethylpyrazine (**1**) and 3,5,6-trimethylpyrazine-2-carboxylic acid (**2**) were prepared according to our previous study [21,22]. In Scheme 1, typical synthetic procedures for **1a**–**5a** involved the combination of bromo TMP and other hydroxybenzoic acids through the formation of ester and ether bonds under alkaline conditions, followed by hydrolysis of the corresponding ligustrazinyloxybenzoic acid esters **1a**–**5a** in 20% (w/v) KOH aqueous solution, to obtain compounds **1b**–**5b**.

*Reagents and Conditions*: (i) DMF, K_2_CO_3_, 85 °C, 1.5 h; (ii) 20% KOH, 60 °C, 45 min.

*Reagents and Conditions*: (i) DMF, NaHCO_3_, r.t., 12 h; (ii) CH_2_Cl_2_, EDCI/DMAP, r.t., 16 h.

In Scheme 2, single ester combination of TMP with hydroxybenzoic acids **1c**–**5c** was achieved by controlling the reaction temperature and alkali catalyst strength. Compounds **1c**–**5c** were reacted with 3,5,6-trimethylpyrazine-2-carboxylic acid (**2**) catalyzed by 1-(3-dimethylaminopropyl)-3-ethylcarbodiimide hydrochloride (EDCI) and 4-dimethylaminopyridine (DMAP) in CH_2_Cl_2_, respectively; we thus successfully obtained compounds **1d**–**5d**. The structures of all the target compounds (Table 1) were confirmed by ^1^H-NMR, ^13^C-NMR, MS and high resolution mass (HRMS). Of all the compounds, **1a**−**5a**, **2b**, **5b**, **1c**−**5c**, **1d**−**5d** were new compounds and **1b**, **3b**, **4b** had been reported previously [11,23], but none of the compounds’ neuroprotective activities had been explored.

**Table 1:** The structures of ligustrazine-benzoic acid derivatives.

Structure	Yield	Structure	Yield
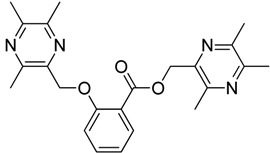	61.1%	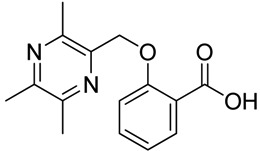	85.1%
**1a**		**1b**	
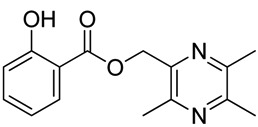	64.0%	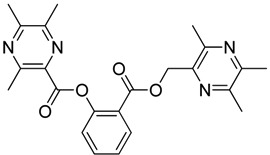	47.6%
**1c**		**1d**	
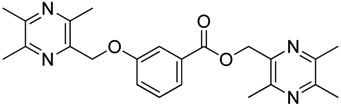	61.7%	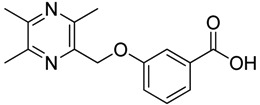	81.9%
**2a**		**2b**	
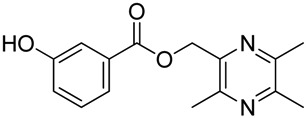	64.4%	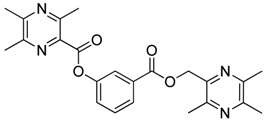	52.6%
**2c**		**2d**	
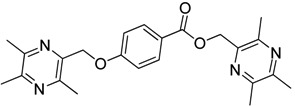	61.7%	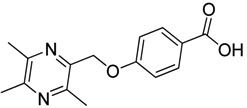	89.7%
**3a**		**3b**	
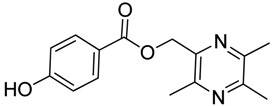	63.2%	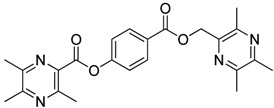	60.1%
**3c**		**3d**	
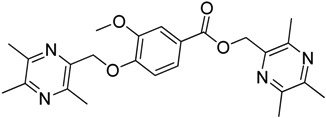	62.5%	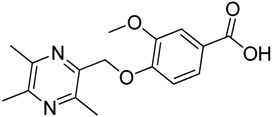	87.4%
**4a**		**4b**	
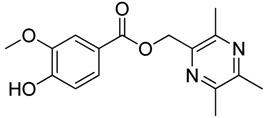	67.5%	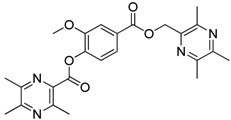	54.7%
**4c**		**4d**	
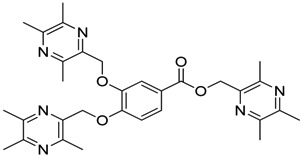	58.4%	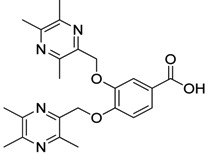	87.1%
**5a**		**5b**	
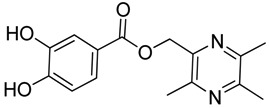	51.2%	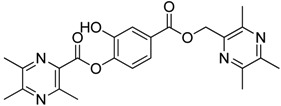	54.7%
**5c**		**5d**	

### 2.2. Biological Activities

#### 2.2.1. Protective Effect on Injured Neuronal-like PC12 Cells

Using TMP as the positive control drug, all the synthesized compounds were tested for their protective effects on neuronal-like PC12 cells damaged by CoCl_2_. This revealed the proliferation rates (%) at different concentration and 50% effective concentrations (EC_50_) for protecting damaged PC12 cells of the ligustrazine-benzoic acid derivatives in Table 2. The results showed that TMP and its derivatives presented protective effects on injured differentiated PC12 cells and most of the ligustrazine derivatives were more active (with lower EC_50_ values) than TMP (EC_50_ = 64.459 µM).

**Table 2:** The EC_50_ of the ligustrazine-benzoic acid derivatives for protecting damaged PC12 cells.

Compound	Proliferation rate (%)					EC_50_ (µM)
	60 µM	30 µM	15 µM	7.5 µM	3.75 µM	
**1a**	61.63	58.87	27.69	18.97	4.58	27.359
**2a**	16.27	25.49	27.48	33.71	15.65	40.715
**3a**	81.60	77.58	64.50	32.08	30.07	11.467
**4a**	126.60	117.10	89.76	52.98	31.12	4.249
**5a**	47.14	36.44	36.01	−7.22	−5.94	45.029
**1b**	5.50	32.37	−1.76	−8.25	−16.00	90.345
**2b**	16.91	27.96	6.99	−22.43	−22.76	95.484
**3b**	27.30	46.91	33.84	11.21	6.44	38.493
**4b**	6.29	10.84	−2.28	−10.40	−10.73	>100
**5b**	27.14	0.47	−0.08	−3.71	−5.67	85.723
**1c**	21.88	21.67	19.32	−1.87	−17.07	73.801
**2c**	55.78	90.74	96.07	72.74	58.09	5.828
**3c**	57.72	18.75	9.20	22.27	−1.86	43.909
**4c**	9.18	12.54	14.28	12.04	7.31	66.230
**5c**	15.68	27.93	27.45	−7.01	−11.72	68.265
**1d**	50.28	41.08	22.15	13.55	2.86	37.398
**2d**	29.07	44.19	37.79	36.63	1.163	33.245
**3d**	46.79	40.13	41.79	36.13	33.47	22.210
**4d**	15.27	62.41	51.74	30.06	1.16	29.683
**5d**	11.50	37.43	23.97	14.19	11.90	46.678
**TMP**	14.71	12.11	11.76	10.60	9.44	64.459

The derivatives containing bis-ligustrazine substituents **1a**–**5a** and **2d**–**5d** exhibited high potency, with EC_50_ values below 50 µM; among them **4a** was the most active compound, with an EC_50_ value of 4.249 µM. We found that most of bis-ligustrazine derivatives’ neuroprotective activities were better than that of the single-ligustrazine componds. This result revealed the combinational effects of two ligustrazinyl pharmacophores. This structure-activity relationship analysis was in agreement with the previously studied protective effects of ligustrazine derivatives on damaged ECV-304 cells [24]. However, this principle did not work in the case of **2c** (EC_50_ = 5.828 µM) on the injured PC12 cells model; this was possibly caused by the usage of different cell types.

In addition, it was observed that ether-joined derivatives’ protective effects were better than those of the ester ones, as exemplified by comparison of the respective pairs of compounds such as **1a** > **1d**, **3a** > **3d**, **4a** > **4d**, **5a** > **5d**. Structure–activity relationship analysis among **1a**–**5a** revealed that compounds with substituents at the *para*-position of the benzoyloxy moiety seemed to be more active than those with substituents at other positions in the same series, and approximately followed an tendency in activity 4-OH, 3-OCH_3_ > 4-OH > 2-OH > 3-OH > 3, 4-OH. These findings may provide a new framework for the design of new ligustrazine derivatives as neuroprotective drugs for treating cerebral ischemic stroke.

Moreover, previous studies displayed that **3b** could inhibit platelet aggregation properties [11,23]; while the current study also showed that **3b** and its congener structures exhibited good neuroprotective activities. Based on the above evidence, we reason that ligustrazine-benzoic acid derivatives possess both thrombolytic and neuroprotective effects and may be more efficacious than either a thrombolytic or neuroprotective agent alone.

#### 2.2.2. Effect of **4a** on CoCl_2_-Induced Cell Injury

**Figure 1:**
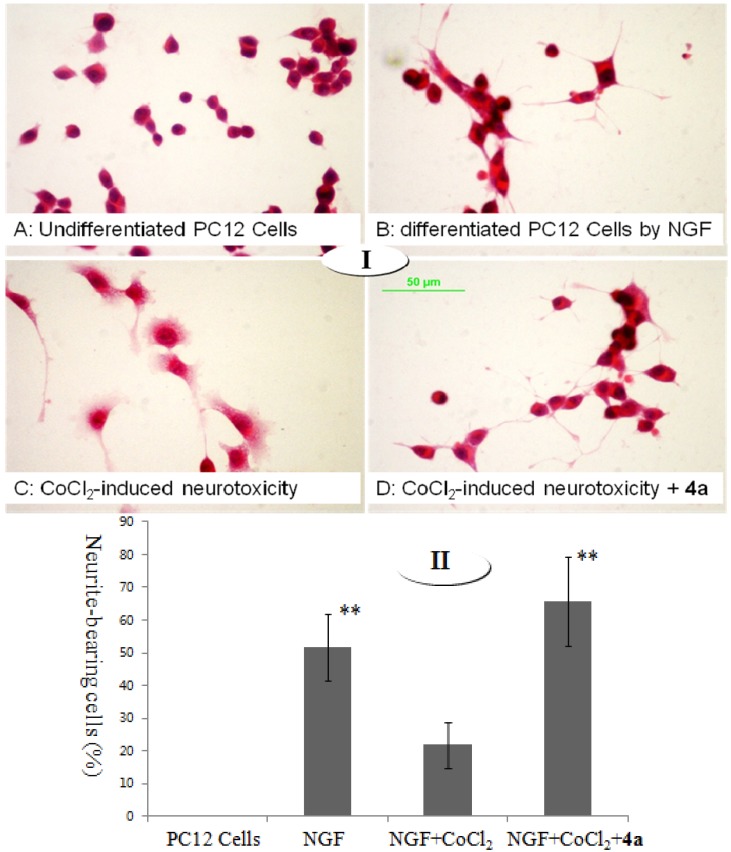
Effect of **4a** on differentiated PC12 cell injury induced by CoCl_2_.

Under optical microscopy, we found that undifferentiated PC12 cells proliferated to form clone-like cell clusters without neural characteristics as shown in Figure 1-I-A; normal differentiated PC12 cells showed round cell bodies with fine dendritic networks, and the cell edges were intact and clear (Figure 1-I-B); moreover, the mean value expressed as percent of neurite-bearing cells in NGF treated cells was 51.7% (Figure 1-II). In contrast, incubation of cells with 200 mM of CoCl_2_ for 12 h induced shrinkage of the cell bodies, disappearance of cell reticular formation, and disruption of the dendritic networks (Figure 1-I-C); the mean value of neurite-bearing cells (21.8%, Figure 1-II) showed a significant decrease. Pretreatment with **4a** dramatically alleviated morphological manifestations of cell damage and led to a pronounced increase (65.8%, Figure 1-II) in neurite-bearing cells compared to model cells (Figure 1-I-D).

## 3. Experimental Section

### 3.1. Chemistry

Reactions were monitored by TLC using silica gel coated aluminum sheets (Qingdao Haiyang Chemical Co., Qingdao, China) and visualized in UV light (254 nm). ^1^H-NMR and ^13^C-NMR assays were recorded on a Bruker AVANCE 500 NMR spectrometer (Fällanden, Switzerland) and chemical shifts are reported in *δ* (ppm). Mass spectra were recorded with an LC Autosampler Device: Standard G1313A instrument (Agilent, New York, NY, USA). HRMS spectra were obtained using a Thermo Scientific^TM^ LTQ Orbitrap XL hybrid FTMS instrument (Thermo Technologies, New York, NY, USA). Melting points (uncorrected) were measured on an X-5 micro melting point apparatus (Beijing, China). Flash column chromatography was performed using 200–300 mesh silica gel. The yields were calculated based on the last step reaction. All chemicals and solvents used were analytical or high-performance liquid chromatography grade.

Values are means ± SD; * *p* < 0.05, ** *p* < 0.01 compared to CoCl_2_ group.

*2-(Bromomethyl)-3,5,6-trimethylpyrazine* (**1**). Compound **1** was prepared according to our previously reported method [21]. The crude product, with 70% purity, was not purified further as it caused a strong mucous membrane irritation.

*3,5,6-Trimethylpyrazine-2-carboxylic acid* (**2**). Compound **2** was prepared according to the method described by Wang *et al.* and Li *et al*., with minor modifications [22,25]. To a solution of trimethylpyrazine (5.0 g, 36.8 mmol) in water (200 mL), aqueous potassium permanganate (KMnO_4_) solution (8.6 g KMnO_4_:150 mL water) was added dropwise at room temperature over about 60 min. Upon completion of the addition, the mixture was stirred at 50 °C for 12 h, then the warm reaction mixture was filtered and washed with hot water (300 mL, 90 °C). The filtrate and washing liquor were combined, cooled to 0–5 °C, and the pH adjusted to 2.0 with concentrated hydrochloric acid. Extraction was performed with ethyl acetate (200 mL × 3), and the organic phase was dried with anhydrous sodium sulfate. The solvent was removed by distillation under vacuum, and the residue was recrystallized from acetone to produce a light yellow solid (2.39 g, yield: 47.8%), m.p.: 162–163 °C [26].

#### 3.1.1. General Procedure for the Preparation of Ligustrazine Derivatives **1a**–**5a** (Scheme 1)

Compound **1** (9.0 mmol) and the corresponding hydroxybenzoic acid (3.0 mmol) were dissolved in dry DMF, then K_2_CO_3_ (6.0 mmol) was added and the mixture was kept at 85 °C for 1.5 h under a nitrogen atmosphere. The warm reaction mixture was poured into ice-water and the crude product was extracted with ethyl acetate. After drying the organic layer over anhydrous Na_2_SO_4_ and evaporating the solvent under vacuum, the crude products were puriﬁed by flash chromatography and recrystallization from acetone.

*(3,5,6-Trimethylpyrazin-2-yl)methyl* *2-[(3,5,6-trimethylpyrazin-2-yl)methoxy]benzoate* (**1a**). White solid, m.p.: 76.2–76.9 °C. ^1^H-NMR (CDCl_3_) (ppm): 7.84 (m, 1H, Ar-H), 7.45 (m, 1H, Ar-H), 7.16 (m, 1H, Ar-H), 6.98 (m, 1H, Ar-H), 5.36 (d, *J* = 2 Hz, 2H, -CH_2_), 5.22 (d, *J* = 2 Hz, 2H, -CH_2_), 2.45–2.52 (m, 18H, -CH_3_). ^13^C-NMR (CDCl_3_) (ppm): 165.8, 158.0, 151.2, 151.2, 150.4, 149.4, 148.8, 148.3, 145.2, 144.9, 133.6, 132.0, 120.6, 120.1, 113.6, 70.7 (-CH_2_), 65.8 (-CH_2_), 21.7 (-CH_3_), 21.7 (-CH_3_), 21.4 (-CH_3_), 21.3 (-CH_3_), 20.6 (-CH_3_), 20.4 (-CH_3_). MS (ESI) *m/z*: 407.1 [M+H]^+^, HRMS (ESI) *m/z*: 407.20633 [M+H]^+^, calcd. for C_23_H_2__7_N_4_O_3_ 407.20049.

*(3,5,6-Trimethylpyrazin-2-yl)methyl* *3-[(3,5,6-trimethylpyrazin-2-yl)methoxy]benzoate* (**2a**)*.* White solid, m.p.: 135.1–135.7 °C. ^1^H-NMR (CDCl_3_) (ppm): 7.70 (brs, 1H, Ar-H), 7.67 (m, 1H, Ar-H), 7.35 (m, 1H, Ar-H), 7.21 (m, 1H, Ar-H), 5.45 (s, 2H, -CH_2_), 5.18 (s, 2H, -CH_2_), 2.51–2.60 (m, 18H, -CH_3_). ^13^C-NMR (CDCl_3_) (ppm): 166.0, 158.6, 151.5, 151.5, 149.9, 149.4, 149.0, 148.8, 145.2, 144.8, 131.1, 129.5, 122.7, 120.2, 115.4, 70.0 (-CH_2_), 66.0 (-CH_2_), 21.7 (-CH_3_), 21.7 (-CH_3_), 21.5 (-CH_3_), 21.4 (-CH_3_), 20.6 (-CH_3_), 20.6 (-CH_3_). MS (ESI) *m/z*: 407.0 [M+H]^+^, HRMS (ESI) *m/z*: 407.20618 [M+H]^+^, calcd. for C_23_H_2__7_N_4_O_3_ 407.20049.

*(3,5,6-Trimethylpyrazin-2-yl)methyl* *4-[(3,5,6-trimethylpyrazin-2-yl)methoxy]benzoate* (**3a**)*.* White solid, m.p.: 70.2–71.0 °C. ^1^H-NMR (CDCl_3_) (ppm): 8.00 (d, *J* = 8.5 Hz, 2H, Ar-H), 7.02 (d, *J* = 9.0 Hz, 2H, Ar-H), 5.42 (s, 2H, -CH_2_), 5.21 (s, 2H, -CH_2_), 2.52-2.59 (m, 18H, -CH_3_). ^13^C-NMR (CDCl_3_) (ppm): 165.9, 162.5, 151.6, 151.3, 150.0, 149.4, 149.0, 148.7, 145.1, 145.0, 131.8, 122.6, 114.5, 70.0 (-CH_2_), 65.7 (-CH_2_), 21.7 (-CH_3_), 21.7 (-CH_3_), 21.5 (-CH_3_), 21.4 (-CH_3_), 20.6 (-CH_3_), 20.6 (-CH_3_). MS (ESI) *m/z*: 407.0 [M+H]^+^, HRMS (ESI) *m/z*: 407.20627 [M+H]^+^, calcd. for C_23_H_2__7_N_4_O_3_ 407.20049.

*(3,5,6-Trimethylpyrazin-2-yl)methyl* *3-methoxy-4-[(3,5,6-trimethylpyrazin-2-yl)methoxy] benzoate* (**4a**)*.* White solid, m.p.: 108.8–109.6 °C. ^1^H-NMR (CDCl_3_) (ppm): 7.64 (dd, *J* = 8.5, 1.5 Hz, 1H, Ar-H), 7.55 (d, *J* = 1.5 Hz, 1H, Ar-H), 7.06 (d, *J* = 8.5 Hz, 1H, Ar-H), 5.43 (s, 2H, -CH_2_), 5.27 (s, 2H, -CH_2_), 3.88 (s, 3H, -OCH_3_), 2.52-2.62 (m, 18H, -CH_3_). ^13^C-NMR (CDCl_3_) (ppm): 166.0, 152.1, 151.3, 151.2, 150.0, 149.2, 149.2, 149.1, 148.7, 145.2, 145.2, 123.6, 122.8, 112.7, 112.6, 70.7 (-CH_2_), 65.7 (-CH_2_), 56.0 (-OCH_3_), 21.6 (-CH_3_), 21.6 (-CH_3_), 21.5 (-CH_3_), 21.4 (-CH_3_), 20.6 (-CH_3_), 20.6 (-CH_3_). MS (ESI) *m/z*: 437.2 [M+H]^+^, HRMS (ESI) *m/z*: 437.21692 [M+H]^+^, calcd. for C_24_H_2__9_N_4_O_4_ 437.21106.

*(3,5,6-Trimethylpyrazin-2-yl)methyl* *3,4-bis[(3,5,6-trimethylpyrazin-2-yl)methoxy]benzoate* (**5a**)*.* White solid, m.p.: 132.9–133.7 °C. ^1^H-NMR (CDCl_3_) (ppm): 7.76 (brs, 1H, Ar-H), 7.69 (d, *J* = 8.5 Hz, 1H, Ar-H), 7.05 (d, *J* = 8.5 Hz, 1H, Ar-H), 5.43 (s, 2H, -CH_2_), 5.23 (s, 2H, -CH_2_), 5.17 (s, 2H, -CH_2_), 2.47–2.60 (m, 27H, -CH_3_). ^13^C-NMR (CDCl_3_) (ppm): 165.8, 152.8, 151.3, 151.3, 151.1, 150.1, 150.0, 149.3, 149.0, 148.6, 148.6, 148.1, 145.4, 145.1, 145.1, 124.5, 122.8, 115.4, 113.1, 71.1 (-CH_2_), 70.8 (-CH_2_), 65.8 (-CH_2_), 21.7 (-CH_3_), 21.7 (-CH_3_), 21.6 (-CH_3_), 21.5 (-CH_3_), 21.3 (-CH_3_), 21.3 (-CH_3_), 20.6 (-CH_3_), 20.6 (-CH_3_), 20.5 (-CH_3_). MS (ESI) *m/z*: 557.2 [M+H]^+^, HRMS (ESI) *m/z*: 557.28503 [M+H]^+^, calcd. for C_31_H_3__7_N_6_O_4_ 557.27980.

#### 3.1.2. General Procedure for the Preparation of Ligustrazine Derivatives **1b**–**5b** (Scheme 1)

An aqueous solution of KOH (4.0 mmol) was added to a solution of ligustrazine derivatives **1a**–**5a** (2.0 mmol) in ethanol (30 mL). The mixture was stirred at 60 °C for 40 min (checked by TLC). Upon completion of the reaction, pH was adjusted to 3 with 10% hydrochloric acid, and then the solution was extracted with ethyl acetate (100 mL). The organic layer was washed with water (100 mL × 2), dried over anhydrous Na_2_SO_4_ and evaporated. The solids were dried under vacuum and purified by recrystallization from acetone to give pure target compounds.

*2-[(3,5,6-Trimethylpyrazin-2-yl)methoxy]benzoic acid* (**1b**). White solid, m.p.: 172.2–172.9 °C. ^1^H-NMR (CDCl_3_) (ppm): 8.10 (d, *J* = 7.5 Hz, 1H, Ar-H), 7.53 (t, *J* = 8.0 Hz, 1H, Ar-H), 7.16 (d, *J* = 8.5 Hz, 1H, Ar-H), 7.12 (t, *J* = 7.5 Hz, 1H, Ar-H), 5.40 (s, 2H, -CH_2_), 2.53 (brs, 6H, -CH_3_), 2.52 (s, 3H, -CH_3_). ^13^C-NMR (CDCl_3_) (ppm): 166.2, 157.0, 151.6, 149.1, 146.9, 143.6, 134.4, 133.6, 122.6, 122.6, 113.6, 68.1 (-CH_2_), 21.5 (-CH_3_), 20.7 (-CH_3_), 20.0 (-CH_3_). MS (ESI) *m/z*: 273.0 [M+H]^+^, HRMS (ESI) *m/z*: 273.12250 [M+H]^+^, calcd. for C_15_H_1__7_N_2_O_3_ 273.11609.

*3-[(3,5,6-Trimethylpyrazin-2-yl)methoxy]benzoic acid* (**2b**). White solid, m.p.: 139.2–139.9 °C. ^1^H-NMR (CDCl_3_) (ppm): 7.79 (brs, 1H, Ar-H), 7.74 (m, 1H, Ar-H), 7.39 (m, 1H, Ar-H), 7.25 (m, 1H, Ar-H), 5.25 (s, 2H, -CH_2_), 2.64 (s, 3H, -CH_3_), 2.57 (brs, 6H, -CH_3_). ^13^C-NMR (CDCl_3_) (ppm): 170.7, 158.5, 151.5, 149.9, 149.0, 145.4, 131.2, 129.6, 123.1, 120.7, 115.6, 69.7 (-CH_2_), 21.5 (-CH_3_), 21.3 (-CH_3_), 20.4 (-CH_3_). MS (ESI) *m/z*: 273.0 [M+H]^+^, HRMS (ESI) *m/z*: 273.12234 [M+H]^+^, calcd. for C_15_H_1__7_N_2_O_3_ 273.11609.

*4-[(3,5,6-Trimethylpyrazin-2-yl)methoxy]benzoic acid* (**3b**). White solid, m.p.: 160.7–161.4 °C. ^1^H-NMR (CDCl_3_) (ppm): 8.07 (d, *J* = 8.5 Hz, 2H, Ar-H), 7.07 (d, *J* = 8.5 Hz, 2H, Ar-H), 5.26 (s, 2H, -CH_2_), 2.63 (s, 3H, -CH_3_), 2.56 (s, 3H, -CH_3_), 2.56 (s, 3H, -CH_3_). ^13^C-NMR (CDCl_3_) (ppm): 171.2, 162.9, 151.6, 150.0, 148.9, 145.1, 132.3, 122.4, 114.6, 69.9 (-CH_2_), 21.7 (-CH_3_), 21.4 (-CH_3_), 20.5 (-CH_3_). MS (ESI) *m/z*: 273.0 [M+H]^+^, HRMS (ESI) *m/z*: 273.12234 [M+H]^+^, calcd. for C_15_H_1__7_N_2_O_3_ 273.11609.

*3-Methoxy-4-[(3,5,6-trimethylpyrazin-2-yl)methoxy]benzoic acid* (**4b**). White solid, m.p.: 190.1–190.9 °C. ^1^H-NMR (CDCl_3_) (ppm): 7.74 (dd, *J* = 8.5, 1.5 Hz, 1H, Ar-H), 7.61 (d, *J* = 1.5 Hz, 1H, Ar-H), 7.12 (d, *J* = 8.5 Hz, 1H, Ar-H), 5.31 (s, 2H, -CH_2_), 3.92 (s, 3H, -OCH_3_), 2.65 (s, 3H, -CH_3_), 2.54 (s, 3H, -CH_3_), 2.55 (s, 3H, -CH_3_). ^13^C-NMR (CDCl_3_) (ppm): 171.2, 152.5, 151.5, 150.1, 149.2, 148.8, 145.1, 124.2, 122.5, 112.7, 112.6, 70.6 (-CH_2_), 56.0 (-OCH_3_), 21.6 (-CH_3_), 21.3 (-CH_3_), 20.5 (-CH_3_). MS (ESI) *m/z*: 303.2 [M+H]^+^, HRMS (ESI) *m/z*: 303.13272 [M+H]^+^, calcd. for C_16_H_1__9_N_2_O_4_ 303.12666.

*3,4-bis[(3,5,6-Trimethylpyrazin-2-yl)methoxy]benzoic acid* (**5b**). White solid, m.p.: 183.8–184.5 °C. ^1^H-NMR (CDCl_3_) (ppm): 7.76 (d, *J* = 1.7 Hz, 1H, Ar-H), 7.69 (dd, *J* = 8.5, 1.7 Hz, 1H, Ar-H), 7.13 (d, *J* = 8.5 Hz, 1H, Ar-H), 5.27 (s, 2H, -CH_2_), 5.24 (s, 2H, -CH_2_), 2.53–2.58 (m, 18H, -CH_3_). ^13^C-NMR (CDCl_3_) (ppm): 170.4, 153.1, 151.5, 151.4, 150.2, 150.2, 148.7, 148.6, 148.0, 145.4, 145.1, 124.9, 122.8, 115.7, 113.0, 70.9 (-CH_2_), 70.7 (-CH_2_), 21.6 (-CH_3_), 21.6 (-CH_3_), 21.3 (-CH_3_), 21.2 (-CH_3_), 20.5 (-CH_3_), 20.5 (-CH_3_). MS (ESI) *m/z*: 423.0 [M+H]^+^, HRMS (ESI) *m/z*: 423.20093 [M+H]^+^, calcd. for C_23_H_2__7_N_4_O_4_ 423.19541.

#### 3.1.3. General Procedure for the Preparation of Ligustrazine Derivatives **1c**–**5c** (Scheme 2)

Compound **1** (3.0 mmol) and hydroxybenzoic acid (3.0 mmol) were dissolved in dry DMF (25 mL), then NaHCO_3_ (4.0 mmol) was added and the mixture was kept at room temperature for 12 h under nitrogen atmosphere. Then reaction mixture was poured into ice-water and the crude product was extracted with ethyl acetate. After drying the organic layer over anhydrous Na_2_SO_4_ and evaporating the solvent under vacuum, the crude products were purified by flash chromatography and recrystallization from acetone.

*(3,5,6-Trimethylpyrazin-2-yl)methyl* *2-hydroxybenzoate* (**1c**). White solid, m.p.: 84.4–85.1 °C. ^1^H-NMR (CDCl_3_) (ppm): 10.66 (s, 1H, -OH), 7.85 (dd, *J* = 8.0, 1.5 Hz, 1H, Ar-H), 7.47 (td, *J* = 8.0, 1.5 Hz, 1H, Ar-H), 7.01 (d, *J* = 8.0 Hz, 1H, Ar-H), 6.87 (t, *J* = 8.0 Hz, 1H, Ar-H), 5.49 (s, 2H, -CH_2_), 2.61 (s, 3H, -CH_3_), 2.56 (s, 3H, -CH_3_), 2.54 (s, 3H, -CH_3_). ^13^C-NMR (CDCl_3_) (ppm): 169.5, 161.6, 151.7, 149.2, 149.1, 144.2, 135.9, 130.1, 119.2, 117.7, 112.3, 65.8 (-CH_2_), 21.7 (-CH_3_), 21.4 (-CH_3_), 20.6 (-CH_3_). MS (ESI) *m/z*: 273.2 [M+H]^+^, HRMS (ESI) *m/z*: 273.12241 [M+H]^+^, calcd. for C_15_H_1__7_N_2_O_3_ 273.11609.

*(3,5,6-Trimethylpyrazin-2-yl)methyl* *3-hydroxybenzoate* (**2c**). White solid, m.p.: 110.1–110.8 °C. ^1^H-NMR (CDCl_3_) (ppm): 7.53 (m, 1H, Ar-H), 7.46 (brs, 1H, Ar-H), 7.26 (m, 1H, Ar-H), 7.04 (m, 1H, Ar-H), 5.42 (s, 2H, -CH_2_), 2.59 (s, 3H, -CH_3_), 2.55 (s, 3H, -CH_3_), 2.53 (s, 3H, -CH_3_). ^13^C-NMR (CDCl_3_) (ppm): 166.2, 156.6, 151.7, 149.5, 149.2, 144.9, 129.7, 121.6, 121.6, 120.9, 116.3, 65.6 (-CH_2_), 21.4 (-CH_3_), 21.2 (-CH_3_), 20.3 (-CH_3_). MS (ESI) *m/z*: 273.0 [M+H]^+^, HRMS (ESI) *m/z*: 273.12201 [M+H]^+^, calcd. for C_15_H_1__7_N_2_O_3_ 273.11609.

*(3,5,6-Trimethylpyrazin-2-yl)methyl* *4-hydroxybenzoate* (**3c**). White solid, m.p.: 183.2–184.0 °C. ^1^H-NMR (DMSO-*d*_6_) (ppm): 10.39 (s, 1H, -OH), 7.81 (d, *J* = 8.5 Hz, 2H, Ar-H), 6.84 (d, *J* = 8.5 Hz, 2H, Ar-H), 5.34 (s, 2H, -CH_2_), 2.50 (s, 3H, -CH_3_), 2.47 (s, 3H, -CH_3_), 2.45 (s, 3H, -CH_3_). ^13^C-NMR (DMSO-*d*_6_) (ppm): 165.7, 162.6, 151.3, 149.2, 148.9, 145.4, 132.1, 120.3, 115.9, 65.3 (-CH_2_), 21.7 (-CH_3_), 21.5 (-CH_3_), 20.6 (-CH_3_). MS (ESI) *m/z*: 273.1 [M+H]^+^, HRMS (ESI) *m/z*: 273.12231 [M+H]^+^, calcd. for C_15_H_1__7_N_2_O_3_ 273.11609.

*(3,5,6-Trimethylpyrazin-2-yl)methyl* *4-hydroxy-3-methoxybenzoate* (**4c**). White solid, m.p.: 147.8–148.4 °C. ^1^H-NMR (CDCl_3_) (ppm): 7.62 (dd, *J* = 8.4, 1.5 Hz, 1H, Ar-H), 7.54 (d, *J* = 1.5 Hz, 1H, Ar-H), 6.90 (d, *J* = 8.4 Hz, 1H, Ar-H), 5.41 (s, 2H, -CH_2_), 3.91 (s, 3H, -OCH_3_), 2.58 (s, 3H, -CH_3_), 2.52 (s, 3H, -CH_3_), 2.51 (s, 3H, -CH_3_). ^13^C-NMR (CDCl_3_) (ppm): 166.0, 151.3, 150.3, 149.3, 149.0, 146.2, 145.1, 124.4, 121.7, 114.1, 111.8, 65.6 (-CH_2_), 56.1 (-OCH_3_), 21.6 (-CH_3_), 21.4 (-CH_3_), 20.6 (-CH_3_). MS (ESI) *m/z*: 303.1 [M+H]^+^, HRMS (ESI) *m/z*: 303.13297 [M+H]^+^, calcd. for C_16_H_1__9_N_2_O_4_ 303.12666.

*(3,5,6-Trimethylpyrazin-2-yl)methyl* *3,4-dihydroxybenzoate* (**5c**). White solid, m.p.: 184.8–185.5 °C. ^1^H-NMR (DMSO-*d*_6_) (ppm): 9.86 (s, 1H, -OH), 9.42 (s, 1H, -OH), 7.35 (brs, 1H, Ar-H), 7.31 (m, 1H, Ar-H), 6.81 (d, *J* = 8.0 Hz, 1H, Ar-H), 5.32 (s, 2H, -CH_2_), 2.50 (s, 3H, -CH_3_), 2.46 (s, 3H, -CH_3_), 2.43 (s, 3H, -CH_3_). ^13^C-NMR (DMSO-*d*_6_) (ppm): 165.8, 151.4, 151.1, 149.2, 148.9, 145.6, 145.4, 122.5, 120.6, 116.7, 115.9, 65.3 (-CH_2_), 21.7 (-CH_3_), 21.5 (-CH_3_), 20.6 (-CH_3_). MS (ESI) *m/z*: 289.1 [M+H]^+^, HRMS (ESI) *m/z*: 289.11725 [M+H]^+^, calcd. for C_15_H_1__7_N_2_O_4_ 289.11101.

#### 3.1.4. General Procedure for the Preparation of Ligustrazine Derivatives **1d**–**5d** (Scheme 2)

To a solution of ligustrazine derivatives **1c**–**5c** (1.0 mmol) in CH_2_Cl_2_ (30 mL) were successively added compound **2** (1.2 mmol), EDCI (1.5 mmol), and DMAP (catalytic amount), and the mixture was stirred at room temperature for 16 h under nitrogen atmosphere. The organic layer was washed with brine and water, respectively. After drying the organic layer over anhydrous Na_2_SO_4_ and evaporating the solvent under vacuum, the crude products were purified by flash chromatography and the target compound was recrystallized from acetone.

*2-[[(3,5,6-Trimethylpyrazin-2-yl)methoxy]carbonyl]phenyl 3,5,6-trimethylpyrazine-2-carboxylate* (**1d**). White solid, m.p.: 89.4–90.1 °C. ^1^H-NMR (CDCl_3_) (ppm): 8.16 (m, 1H, Ar-H), 7.63 (m, 1H, Ar-H), 7.39 (m, 1H, Ar-H), 7.26 (m, 1H, Ar-H), 5.30 (s, 2H, -CH_2_), 2.68 (s, 3H, -CH_3_), 2.63 (brs, 6H, -CH_3_), 2.45 (brs, 6H, -CH_3_), 2.42 (s, 3H, -CH_3_). ^13^C-NMR (CDCl_3_) (ppm): 164.3, 164.1, 155.2, 153.0, 151.3, 151.0, 149.5, 149.5, 148.7, 144.2, 137.3, 134.2, 132.4, 126.4, 124.2, 123.2, 66.3 (-CH_2_), 23.0 (-CH_3_), 22.5 (-CH_3_), 21.7 (-CH_3_), 21.7 (-CH_3_), 21.3 (-CH_3_), 20.4 (-CH_3_). MS (ESI) *m/z*: 421.0 [M+H]^+^, HRMS (ESI) *m/z*: 421.18494 [M+H]^+^, calcd. for C_23_H_2__5_N_4_O_4_ 421.17976.

*3-[[(3,5,6-Trimethylpyrazin-2-yl)methoxy]carbonyl]phenyl* *3,5,6-trimethylpyrazine-2-carboxylate* (**2d**)*.* White solid, m.p.: 135.1–135.7 °C. ^1^H-NMR (CDCl_3_) (ppm): 7.97 (m, 1H, Ar-H), 7.91 (brs, 1H, Ar-H), 7.50 (m, 1H, Ar-H), 7.45 (m, 1H, Ar-H), 5.44 (s, 2H, -CH_2_), 2.81 (s, 3H, -CH_3_), 2.61 (s, 3H, -CH_3_), 2.61 (s, 3H, -CH_3_), 2.58 (s, 3H, -CH_3_), 2.51 (s, 3H, -CH_3_), 2.50 (s, 3H, -CH_3_). ^13^C-NMR (CDCl_3_) (ppm): 165.2, 164.2, 155.5, 152.7, 151.5, 150.8, 149.7, 149.3, 149.1, 144.6, 137.9, 131.4, 129.5, 127.5, 126.8, 123.3, 66.1 (-CH_2_), 22.9 (-CH_3_), 22.3 (-CH_3_), 21.7 (-CH_3_), 21.6 (-CH_3_), 21.5 (-CH_3_), 20.6 (-CH_3_). MS (ESI) *m/z*: 421.0 [M+H]^+^, HRMS (ESI) *m/z*: 421.18512 [M+H]^+^, calcd. for C_23_H_2__5_N_4_O_4_ 421.17976.

*4-[[(3,5,6-Trimethylpyrazin-2-yl)methoxy]carbonyl]phenyl 3,5,6-trimethylpyrazine-2-carboxylate* (**3d**)*.* White solid, m.p.: 89.5–90.2 °C. ^1^H-NMR (CDCl_3_) (ppm): 8.14 (d, *J* = 8.5 Hz, 2H, Ar-H), 7.32 (d, *J* = 9.0 Hz, 2H, Ar-H), 5.46 (s, 2H, -CH_2_), 2.83 (s, 3H, -CH_3_), 2.63 (s, 3H, -CH_3_), 2.63 (s, 3H, -CH_3_), 2.60 (s, 3H, -CH_3_), 2.54 (s, 3H, -CH_3_), 2.52 (s, 3H, -CH_3_). ^13^C-NMR (CDCl_3_) (ppm): 165.4, 163.9, 155.7, 154.7, 152.8, 151.5, 149.7, 149.3, 149.1, 144.7, 137.8, 131.4, 127.6, 122.0, 66.0 (-CH_2_), 22.9 (-CH_3_), 22.4 (-CH_3_), 21.7 (-CH_3_), 21.7 (-CH_3_), 21.5 (-CH_3_), 20.6 (-CH_3_). MS (ESI) *m/z*: 421.0 [M+H]^+^, HRMS (ESI) *m/z*: 421.18521 [M+H]^+^, calcd. for C_23_H_2__5_N_4_O_4_ 421.17976.

*2-Methoxy-4-[[(3,5,6-trimethylpyrazin-2-yl)methoxy]carbonyl]phenyl* *3,5,6-trimethylpyrazine-2-carboxylate* (**4d**). White solid, m.p.: 113.2–113.8 °C. ^1^H-NMR (CDCl_3_) (ppm): 7.73 (m, 1H, Ar-H), 7.72 (brs, 1H, Ar-H), 7.26 (d, *J* = 8.0 Hz, 1H, Ar-H), 5.46 (s, 2H, -CH_2_), 3.88 (s, 3H, -OCH_3_), 2.84 (s, 3H, -CH_3_), 2.64 (s, 3H, -CH_3_), 2.62 (s, 3H, -CH_3_), 2.61 (s, 3H, -CH_3_), 2.54 (s, 3H, -CH_3_), 2.53 (s, 3H, -CH_3_). ^13^C-NMR (CDCl_3_) (ppm): 165.6, 163.4, 155.4, 152.7, 151.5, 151.1, 149.7, 149.3, 149.1, 144.7, 144.0, 138.0, 128.7, 123.0, 122.9, 113.6, 66.0 (-CH_2_), 56.1 (-OCH_3_), 22.8 (-CH_3_), 22.4 (-CH_3_), 21.7 (-CH_3_), 21.7 (-CH_3_), 21.5 (-CH_3_), 20.6 (-CH_3_). MS (ESI) *m/z*: 451.2 [M+H]^+^, HRMS (ESI) *m/z*: 451.19550 [M+H]^+^, calcd. for C_24_H_2__7_N_4_O_5_ 451.19032.

*2-Hydroxy-4-[[(3,5,6-trimethylpyrazin-2-yl)methoxy]carbonyl]phenyl* *3,5,6-trimethylpyrazine-2-carboxylate* (**5d**)*.* White solid, m.p.: 182.2–182.9 °C. ^1^H-NMR (CDCl_3_) (ppm): 9.70 (brs, 1H, -OH), 7.88 (s, 1H, Ar-H), 7.83 (m, 1H, Ar-H), 7.01 (d, *J* = 8.5 Hz, 1H, Ar-H), 5.40 (s, 2H, -CH_2_), 2.72 (s, 3H, -CH_3_), 2.58 (brs, 6H, -CH_3_), 2.53 (s, 3H, -CH_3_), 2.51 (s, 3H, -CH_3_), 2.50 (s, 3H, -CH_3_). ^13^C-NMR (CDCl_3_) (ppm): 165.3, 163.2, 156.2, 153.2, 152.8, 151.3, 149.6, 149.3, 149.1, 145.1, 137.9, 137.2, 129.6, 125.1, 121.7, 117.4, 65.5 (-CH_2_), 22.5 (-CH_3_), 22.2 (-CH_3_), 21.5 (-CH_3_), 21.3 (-CH_3_), 21.2 (-CH_3_), 20.5 (-CH_3_). MS (ESI) *m/z*: 437.2 [M+H]^+^, HRMS (ESI) *m/z*: 437.18036 [M+H]^+^, calcd. for C_2__3_H_2__5_N_4_O_5_ 437.17467.

### 3.2. Bio-Evaluation Methods

#### 3.2.1. Protective Effect on Damaged Differentiated PC12 Cells [9,24,25,26]

PC12 cells were cultured in RPMI 1640 medium supplemented with 5% (v/v) fetal bovine serum, 10% (v/v) horse serum and 100 U/mL penicillin-streptomycin (Thermo Technologies, New York, NY, USA) at 37 °C in a humidified atmosphere of 5% CO_2_. When cells achieved the desired density of >80% confluency original medium was removed and cells were cultured with the serum-free medium for 14 h. Then the cells were suspended in 1640 medium supplemented with 10% (v/v) fetal bovine serum, and seeded into poly-L-lysine-coated 96-well culture plates at 7 × 10^3^ cells/well, differentiated by treated with 50 ng/mL NGF for 48 h. After these, the differentiated PC12 cells were pretreated with various concentrations (60, 30, 15, 7.5, 3.75 µM) of ligustrazine derivatives for 36 h. All measurements were performed after the cells were induced by CoCl_2_ (final concentration, 200 mM) for 12 h. Control differentiated cells were not treated with ligustrazine derivatives and CoCl_2_. CoCl_2_ was dissolved in RPMI 1640 medium. ligustrazine derivatives were dissolved in DMSO. The final concentration of DMSO was less than 0.1% (v/v).

After MTT solution (20 µL, 5 mg/mL) was added to each well, the plate was incubated for a further 4 h at 37 °C. The supernatant was removed carefully by pipetting from wells without disturbing the attached cells and formazan crystals were solubilized by adding 200 µL of DMSO to each well and shaken for 15 min. The absorbance at 490 nm was measured with a BIORAD 550 spectrophotometer (Bio-rad, California, CA, USA). The proliferation rates of damaged PC12 cells were calculated by the formula [OD_490_ (Compd) − OD_490_ (CoCl_2_)]/[OD_490_ (NGF) − OD_490_ (CoCl_2_)] × 100%; the EC_50_ values were defined as the concentration of compounds that produced a 50% proliferation of surviving cells and calculated using the following equation: −pEC_50_ = log C_max_ − log 2 × (∑P − 0.75 + 0.25P_max_ + 0.25P_min_), Where C_max_ = maximum concentration, ∑P = sum of proliferation rates, P_max_ = maximum value of proliferation rate and P_min_ = minimum value of proliferation rate.

#### 3.2.2. Observation of Morphologic Changes [26,27]

The PC12 cells culture procedure was similar to that described in 23. After pretreatment with the serum-free medium for 14 h, cells were seeded at a concentration of 7 × 10^4^ cell/mL in a volume of 0.8 mL on a poly-L-lysine-coated sterile cover slip in 6-well tissue culture plates, and differentiated by treating with NGF (50 ng/mL) for 48 h. Then the differentiated PC12 cells were pretreated with compound **4a** (60 µM) for 36 h prior to exposure to CoCl_2_. After induction by CoCl_2_ for 12 h, culture medium was removed and the cells were fixed in 95% ethyl alcohol for 15 min. The cells were washed twice with PBS and stained with hematoxylin for 8 min. The stained nuclei were washed twice with diluted PBS, and consequently stained with eosin for 8 min. Next, H&E stained sections were dehydrated through gradient ethanol and cleared in xylene. The residual procedure was processed in accordance with the hematoxylin and eosin (HE) staining protocol. The cellular morphology and percentage of cells showing neurite outgrowth was determined by light microscopy (Nikon, Kobe, Japan). Cells with one or more neurites whose lengths were at least twice the diameter of the cell body were scored as differentiated cells. Cell differentiation rate was calculated as the number of differentiated cells/total cells. Neurite outgrowth was determined from at least three different regions of interest in three independent experiments. All data are expressed as mean ± SD. Data analysis was carried out using SAS software, version 8.1 (SAS Institute, Cary, NC, USA). Statistically significant differences between the samples were evaluated by Student’s t-test and *p* < 0.05 was considered significant.

## 4. Conclusions

In conclusion, a series of novel ligustrazine-benzoic acid derivatives was designed, synthesized and biologically evaluated for their protective effects on the damaged differentiated PC12 cell proliferation. The preliminary biological results have demonstrated that most of ligustrazine-benzoic acid derivatives exhibited good protective effects in comparison with TMP. Among the active compounds, **4a** is the most active congener, with EC_50_ 4.249 µM, which is much higher than that of TMP, and representing a most promising lead for further investigation. Studies of the anti-experimental stroke effects and mechanism of **4a** in a rat middle cerebral artery occlusion (MCAO) stroke model are in progress and will be reported in the near future.

## Supplementary Materials

Supplementary materials can be accessed at: http://www.mdpi.com/1420-3049/18/10/13027/s1.
